# The Association of Unfavorable Outcomes with the Intensity of Neurosurgical Care in the United States

**DOI:** 10.1371/journal.pone.0092057

**Published:** 2014-03-19

**Authors:** Symeon Missios, Kimon Bekelis

**Affiliations:** 1 Department Neurosurgery, Cleveland Clinic, Cleveland, Ohio, United States of America; 2 Section of Neurosurgery, Dartmouth-Hitchcock Medical Center, Lebanon, New Hampshire, United States of America; Johns Hopkins Hospital, United States of America

## Abstract

**Object:**

There is wide regional variability in the volume of procedures performed for similar surgical patients throughout the United States. We investigated the association of the intensity of neurosurgical care (defined as the average annual number of neurosurgical procedures per capita) with mortality, length of stay (LOS), and rate of unfavorable discharge for inpatients after neurosurgical procedures.

**Methods:**

We performed a retrospective cohort study involving the 202,518 patients who underwent cranial neurosurgical procedures from 2005–2010 and were registered in the National Inpatient Sample (NIS) database. Regression techniques were used to investigate the association of the average intensity of neurosurgical care with the average mortality, LOS, and rate of unfavorable discharge.

**Results:**

The inpatient neurosurgical mortality, rate of unfavorable discharge, and average LOS varied significantly among several states. In a multivariate analysis male gender, coverage by Medicaid, and minority racial status were associated with increased mortality, rate of unfavorable discharge, and LOS. The opposite was true for coverage by private insurance, higher income, fewer comorbidities and small hospital size. There was no correlation of the intensity of neurosurgical care with the mortality (Pearson's ρ = −0.18, P = 0.29), rate of unfavorable discharge (Pearson's ρ = 0.08, P = 0.62), and LOS of cranial neurosurgical procedures (Pearson's ρ = −0.21, P = 0.22).

**Conclusions:**

We observed significant disparities in mortality, LOS, and rate of unfavorable discharge for cranial neurosurgical procedures in the United States. Increased intensity of neurosurgical care was not associated with improved outcomes.

## Introduction

The implementation of accountable care will bring a paradigm shift in physician reimbursement, from the established pay-for-service to the new pay-for-performance model [1], [2]. This will attempt to address the reality that increased volume of procedures does not always correlate with higher quality of healthcare delivery [1], [2]. In surgery, in particular, practice patterns vary widely throughout the United States for different interventions [3]–[5]. The rates of procedures performed on similar patients are tremendously different for separate regions [3]–[5]. These disparities have been ascribed in part to race and other socioeconomic factors [6]. Prior research in cardiovascular disease [7] has demonstrated that the structural components of hospitals (size, teaching status, financial status) and surgeon characteristics (volume, use of endovascular procedures) may also explain this variation.

This phenomenon has not been previously described in neurosurgery. Several regions might demonstrate different intensity of neurosurgical care (defined as the average annual number of neurosurgical procedures per capital), reflecting local variations in the aggressiveness for intervention. Although increased intensity generates rising costs [3], [4], its correlation with improved outcomes has not been proven. This supports the need to improve quality instead of quantity in the effort to optimize healthcare delivery. The study of this phenomenon will provide actionable information about the quality of specific health-care systems.

In the current study, using the National Inpatient Sample (NIS), we mapped the regional variations in the intensity of neurosurgical care and investigated their association with in-hospital mortality, unfavorable discharge, and length of stay (LOS) in patients undergoing cranial neurosurgical procedures. The NIS is an all-payer and age hospital discharge database that represents approximately 20% of all inpatient admissions to nonfederal hospitals in the United States [8].

## Methods

### National Inpatient Sample (NIS) Database

All patients undergoing cranial neurosurgical interventions in the National Inpatient Sample (NIS) Database [8] (Healthcare Cost and Utilization Project, Agency for Healthcare Research and Quality, Rockville, MD) between 2005 and 2010 were included in the analysis. For these years, the NIS contains discharge data regarding 100% of discharges from a stratified random sample of nonfederal hospitals in several States to approximate a representative 20% subsample of all nonfederal US hospital discharges. More information about the NIS is available at http://www.ahcpr.gov/data/hcup/nisintro.htm.

### Cohort Definition

In order to establish the cohort of patients, we used ICD-9-CM codes to identify patients in the registry who underwent any cranial neurosurgical procedure (craniotomy for aneurysm clipping, craniotomy for tumor resection, craniotomy for AVM resection, craniotomy for epilepsy, shunt placement, craniotomy/burr holes for trauma, deep brain stimulation, transphenoidal pituitary tumor resection) between 2005 and 2010 (Table S1).

### Outcome Variables

The primary outcome variables were the average in-hospital neurosurgical mortality, the average length-of-stay (LOS) for neurosurgical admissions, and the average rate of unfavorable discharge per state per year. Unfavorable discharge was defined as discharge to a facility other than the patient's home (e.g. nursing home, rehab, hospice). National estimates on the number of procedures were created based on the standardized weights provided by the NIS. The population of each state was calculated based on the 2010 US census data.

### Exposure variables

The association of the outcomes with the pertinent exposure variables was examined using regression analysis. Age and neurosurgical intensity were the only 2 continuous variables. Gender, race (African American, Hispanic, Asian, or other, with Caucasian being the reference value), insurance (private insurance, self pay, Medicaid, with Medicare being the reference value), income, and modified Charlson Comorbidity Index (CCI) [9], [10] were categorical variables. Income was defined as the median income based on zip code, and was divided into quartiles, with the lowest quartile being the reference value. The average intensity of neurosurgical care was defined as the average number of neurosurgical procedures (Table S1) performed per capita over a year in a state. Quartiles of intensity were created based on the 25^th^, 50^th^, and 75^th^ percentiles of the distribution of the intensity scores.

The hospital characteristics, used in the analysis as categorical variables, included hospital region (West, South, Midwest, with West being the reference value), hospital location (urban teaching, urban non-teaching, with urban teaching being the reference value), and hospital bed size (medium, large, with large being the reference value). More information of the definitions of the various categories of hospital characteristics can be found at http://www.hcup-us.ahrq.gov/db/vars/nis_stratum/nisnote.jsp.

### Statistical analysis

States with inadequate data on intensity and number of neurosurgical procedures were not included in the analysis. The following states were excluded from the intensity calculation due to lack of data or inadequate data: AK, AL, ND, ME, MS, MT, NM, RI, SD, WY and DE.

Multiple imputation was performed for each variable associated with missing values. This was executed using the multiple imputation suite of commands available in SPSS version 20 (IBM Corp.). Imputation was used for the following missing data: Gender, Age, Payer source, Income and Race. First the proportion of missing data for variables of interest was calculated. The SPSS set of commands was used to generate a regression model to impute missing data based on other available variables. This process was repeated 5 times, creating 5 separate imputed data sets. These 5 data sets were combined to create a full-pooled data set with no missing values, which was used in a multinomial logistic regression model.

A logistic regression model was used to determine the association between mortality and the independent variables. Similarly a logistic regression model was used for unfavorable discharge. A linear regression model was used to analyze the association between the independent variables and the average length of stay. Scatter plots were created and the Pearson correlation coefficients between the intensity of neurosurgical care and the respective primary outcomes were calculated. We compared the rate of primary outcomes between the different states using analysis of variance (ANOVA). No data transformations were employed.

All probability values are the results of two-sided tests, and the level of significance was set at P<0.05. Statistical analyses were performed using the XLSTAT version 2011.6.09 (Addinsoft) and SPSS Statistics version 20 (IBM, Armonk, NY).

## Results

### Demographics and clinical characteristics of the cohort

**Figure 1:**
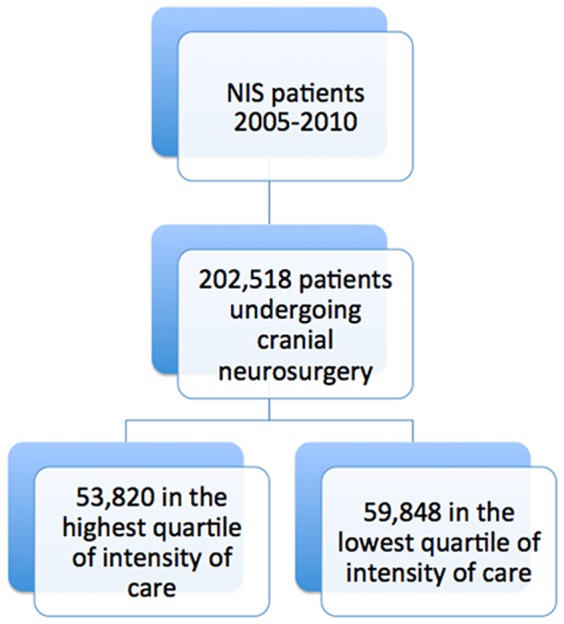
Cohort selection for the study.

In the study period there were 202,518 patients (Figure 1) undergoing cranial neurosurgical procedures (mean age was 56.1 years, with 46.8% females), who were registered in NIS, of whom 53,820 were treated in areas of the highest intensity, and 59,848 were treated in areas of the lowest intensity. Tables 1 and 2 demonstrate the distribution of socioeconomic and other exposure variables among all patients, as well as for patients in the highest and the lowest quartile of neurosurgical intensity. Table 3 demonstrates the outcomes for all patients.

**Table 1:** Patient charactertics.

		All Patients		Top (4^th^) intensity quartile patients		Low (1^st^) intensity quartile patients		P-Value
		**N**		**N**		**N**		
Sample size		202,518		53,820		59,848		
		**Mean**	**SD**	**Mean**	**SD**	**Mean**	**SD**	
Age		56.06	18.13	56.74	18.27	55.99	18.14	<0.0001
		**N**	**%**	**N**	**%**	**N**	**%**	
Sex	F	94,016	46.77	25,172	46.78	27,891	46.62	0.576
	M	106,985	53.23	28,643	53.22	31,940	53.38	0.616
	Unreported data			5		17		
Quartiles of median income based on zip code	1^st^ Quartile	46,890	23.83	12,573	24.50	14,193	24.24	0.160
	2^nd^ Quartile	48,581	24.69	13,926	27.14	14,057	24.01	<0.0001
	3^rd^ Quartile	49,945	25.38	13,316	25.95	14,282	24.39	0.001
	4^th^ Quartile	51,370	26.10	11,499	22.41	16,021	27.36	<0.0001
	Unreported data	5,732		2,506		1,295		
Payer	Medicare	75,450	37.33	20,940	38.92	21,899	36.61	<0.0001
	Medicaid	22,398	11.08	6,315	11.74	5,818	9.73	<0.0001
	Private payer	83,417	41.27	21,213	39.43	24,988	41.77	<0.0001
	Self-payer	11,061	5.47	2,503	4.65	3,994	6.68	<0.0001
	Other	9,784	4.84	2,830	5.26	3,123	5.22	0.762
	Unreported data	408		19		26		
Charlson Comorbidity Index	Low (0-3)	174,358	86.10	45,982	85.44	51,171	85.50	0.756
	Moderate/High (> = 4)	28,160	13.90	7,838	14.56	8,677	14.50	
Race	Caucasian	112,557	71.63	35188	73.00	32,685	69.75	<0.0001
	African American	16,449	10.47	4,604	9.55	6,159	13.14	<0.0001
	Hispanic	16,711	10.64	4,595	9.53	5,084	10.85	0.796
	Asian	5,281	3.36	1,044	2.17	1,092	2.33	0.153
	Other	6,129	3.90	2,775	5.76	1,838	3.92	<0.0001
	Unreported cases	45,391		5,614		12,990		

**Table 2:** Hospital and practice characteristics.

		All Patients		Top (4^th^) intensity quartile patients		Low (1^st^) intensity quartile patients		P-Value
		N	%	N	%	N	%	
Region	West	52,063	25.71	8,975	16.68	8,405	14.04	<0.0001
	South	74,894	36.98	24,007	44.61	26,823	44.82	0.472
	Midwest	42,770	21.12	0	0	20,197	33.75	<0.0001
	Northeast	32,791	16.19	20,838	38.72	4,423	7.39	<0.0001
Location	Urban Teaching	152,408	75.26	40,350	74.97	46,103	77.03	<0.0001
	Urban Nonteaching	45,755	22.59	11,291	20.98	13,023	21.76	0.001
	Rural	4,355	2.15	2,179	4.05	722	1.21	<0.0001
Bedsize	Large	157,945	77.99	45,703	84.92	44,337	74.08	<0.0001
	Medium	33,228	16.41	6,476	12.03	11,411	19.07	<0.0001
	Small	11,345	5.60	1,641	3.05	4,100	6.85	<0.0001
Neurosurgeons per capita	First quartile	50,642	25.01	3,328	6.18	7,068	11.81	<0.0001
	Second quartile	45,918	22.67	3,857	7.17	42,061	70.28	<0.0001
	Third quartile	47,564	23.49	3,653	6.79	8,490	14.19	<0.0001
	Fourth quartile	58,394	28.83	42,982	79.86	2,229	3.72	<0.0001

**Table 3:** Outcomes of patients undergoing cranial neurosurgical procedures in the United States.

	All patients		Highest intensity quartile patients		Lowest intensity quartile patients		P-Value
	**N**	**%**	**N**	**%**	**N**	**%**	
Mortality	15361	7.59	4274	7.94	4369	7.30	0.228
Unfavorable discharge	80112	39.56	22256	41.35	23453	39.19	<0.0001
	**Mean**	**SD**	**Mean**	**SD**	**Mean**	**SD**	
Mean length of stay	10.03	13.35	10.89	15.55	9.93	12.35	<0.0001

### Intensity of neurosurgical care and mortality

**Figure 2:**
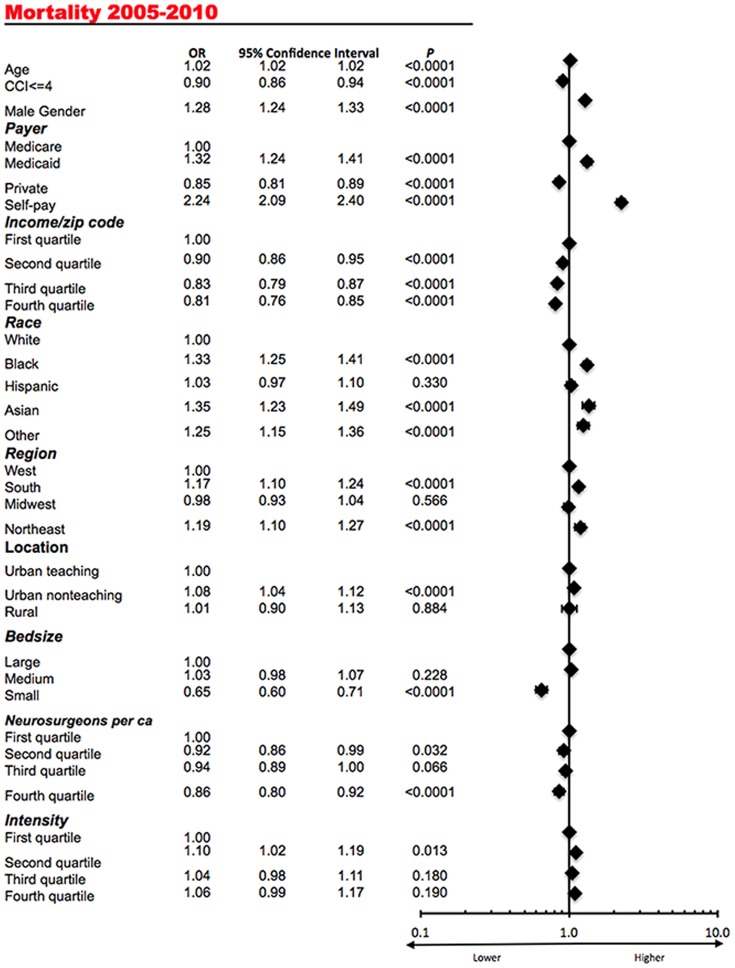
Multivariate analysis demonstrating the association of the exposure variables with the average annual mortality for cranial neurosurgical procedures. The corresponding forest plot is presented on the right.

The in-hospital neurosurgical mortality varied significantly among several states (ANOVA, P<0.0001). In a multivariate analysis (Figure 2) higher age (OR, 1.02; 95% CI, 1.02 to 1.02), male gender (OR, 1.28; 95% CI, 1.24 to 1.33), coverage by Medicaid (OR, 1.32; 95% CI, 1.24 to 1.41), no insurance coverage (OR, 2.24; 95% CI, 2.09 to 2.40), minority racial status (OR, 1.33, 95% CI, 1.25 to1.41 for African Americans in comparison to Caucasian patients), hospital location in the South (OR, 1.17; 95% CI, 1.10 to 1.24 in comparison to the West) and the Northeast (OR, 1.19; 95% CI, 1.10 to 1.27 in comparison to the West), and urban non-teaching hospitals (OR, 1.08; 95% CI, 1.04 to 1.13 in comparison to urban teaching hospitals) were associated with higher mortality. The opposite was true for coverage by private insurance (OR, 0.85; 95% CI, 0.81 to 0.89), higher income (OR, 0.81; 95% CI, 0.76 to 0.85, for the highest quartile in comparison to the lowest quartile), fewer comorbidities (OR, 0.90; 95% CI, 0.86 to 0.94) and small hospital size (OR 0.65; 95% CI, 0.60 to 0.71). Higher number of per capita neurosurgeons did not demonstrate a clear association with improved survival although a trend to that direction was observed. Increasing intensity of neurosurgical care did not demonstrate a clear association with mortality (with most quartiles demonstrating a non-significant association).

**Figure 3:**
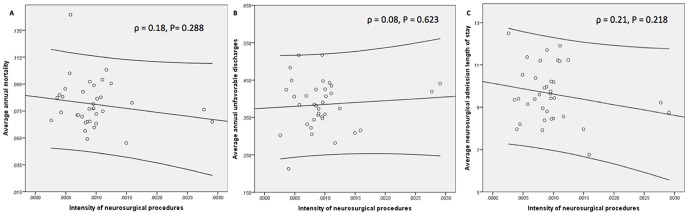
Scatter plot demonstrating the correlation of the average intensity of neurosurgical care with (A) the average annual mortality (Pearson's ρ = −0.18, P = 0.29), (B) the average annual rate of unfavorable discharge (Pearson's ρ = 0.08, P = 0.62), and (C) the average length of stay for cranial neurosurgical procedures (Pearson's ρ = −0.21, P = 0.22).

Overall, as Figure 3A demonstrates, there was no correlation of the average intensity of neurosurgical care and the average annual mortality (Pearson's ρ = −0.18, P = 0.29).

### Intensity of neurosurgical care and unfavorable discharge

**Figure 4:**
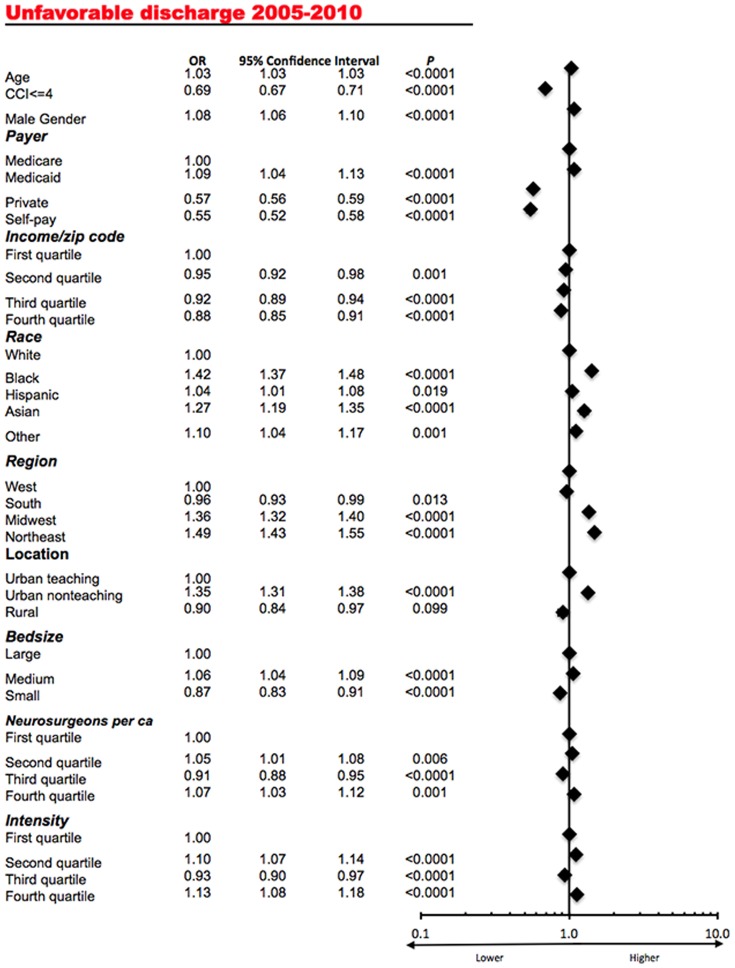
Multivariate analysis demonstrating the association of the exposure variables with the average annual rate of unfavorable discharge for cranial neurosurgical procedures. The corresponding forest plot is presented on the right.

The average annual rate of unfavorable discharge varied significantly among several states (ANOVA, P<0.0001). In a multivariate analysis (Figure 4) higher age (OR, 1.03; 95% CI, 1.03 to 1.03), male gender (OR, 1.08; 95% CI, 1.06 to 1.10), coverage by Medicaid (OR, 1.09; 95% CI, 1.04 to 1.13), minority racial status (OR, 1.42, 95% CI, 1.37 to1.48 for African Americans in comparison to Caucasian patients), hospital location in the Midwest (OR, 1.36; 95% CI, 1.32 to 1.40 in comparison to the West) and the Northeast (OR, 1.49; 95% CI, 1.43 to 1.55 in comparison to the West), and urban non-teaching hospitals (OR, 1.35; 95% CI, 1.31 to 1.38 in comparison to urban teaching hospitals) were associated with higher mortality. The opposite was true for coverage by private insurance (OR, 0.57; 95% CI, 0.56 to 0.59), no insurance coverage (OR, 0.55; 95% CI, 0.52 to 0.58), higher income (OR, 0.88; 95% CI, 0.85 to 0.91, for the highest quartile in comparison to the lowest quartile), fewer comorbidities (OR, 0.69; 95% CI, 0.67 to 0.71), and small hospital size (OR 0.87; 95% CI, 0.83 to 0.91). Higher number of per capita neurosurgeons did not demonstrate a clear direction in its association with the average annual rate of unfavorable discharge. Likewise, increasing intensity of neurosurgical care did not demonstrate a clear association with the rate of unfavorable discharge.

Overall, as Figure 3B demonstrates, there was no correlation of the average intensity of neurosurgical care and the average annual rate of unfavorable discharge (Pearson's ρ = 0.08, P = 0.62).

### Intensity of neurosurgical care and length of stay (LOS)

**Figure 5:**
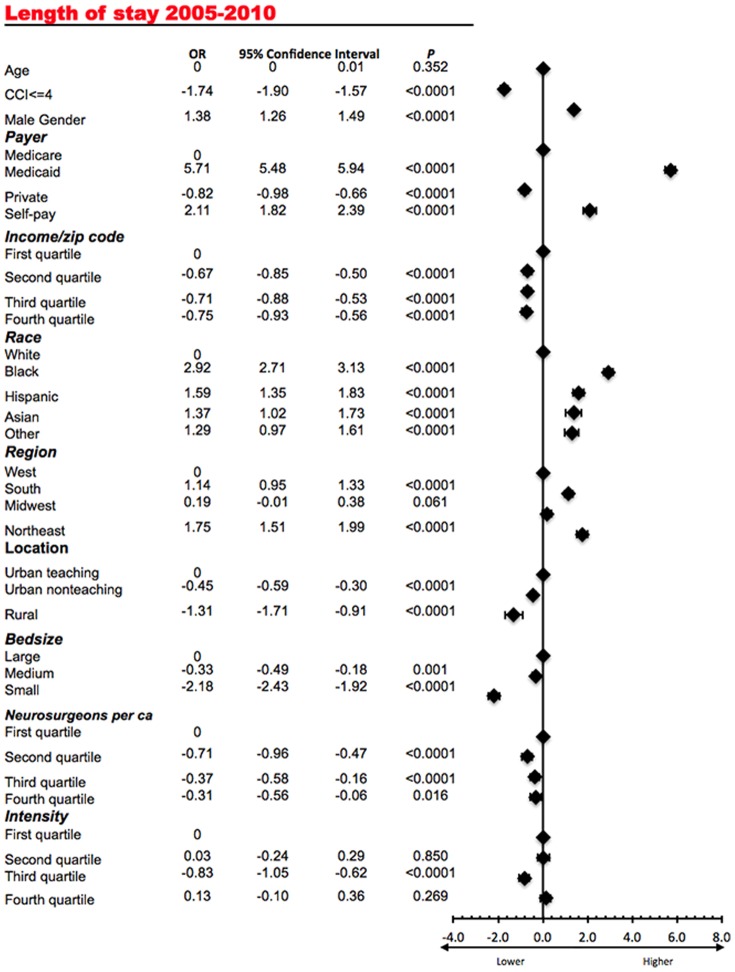
Multivariate analysis demonstrating the association of the exposure variables with the average length of stay for cranial neurosurgical procedures rate. The corresponding forest plot is presented on the right.

The average LOS for a neurosurgical procedure varied significantly among several states (ANOVA, P<0.0001). In a multivariate analysis (Figure 5) male gender (β, 1.38; 95% CI, 1.26 to 1.49), coverage by Medicaid (β, 5.71; 95% CI, 5.48 to 5.94), no insurance coverage (β, 2.11; 95% CI, 1.82 to 2.39), hospital location in the Northeast (β, 1.75; 95% CI, 1.51 to 1.99 in comparison to the West), and minority racial status (β, 2.92; 95% CI, 2.71 to 3.13 for African Americans in comparison to Caucasian patients) were associated with higher LOS. The opposite was true for coverage by private insurance (β, −0.82; 95% CI, −0.98 to −0.66), higher income (β, −0.75; 95% CI, −0.93 to −0.56, for the highest quartile in comparison to the lowest quartile), urban nonteaching hospitals (β, −0.45; 95% CI, −0.59 to −0.30 in comparison to urban teaching), fewer comorbidities (β, −1.74; 95% CI, −1.90 to −1.57), and small hospital size (β, −2.18; 95% CI, −2.43 to −1.92). Higher number of per capita neurosurgeons demonstrated a trend towards decreased LOS. Increasing intensity of neurosurgical care did not demonstrate a clear association with the average LOS (with most quartiles demonstrating a non-significant association).

Overall, as Figure 3C demonstrates, there was no correlation of the average intensity of neurosurgical care and the average LOS for a neurosurgical admission (Pearson's ρ = −0.21, P = 0.22).

## Discussion

Large variations in the use of medical and surgical treatments across hospitals and regions among similar patients have been documented [3], [4], [11]–[13]. In some cases, the association between spending and outcomes is positive [14]–[17], while in others there is no such relationship [3], [11], [13], [18], [19]. The recent emphasis on accountability aims at minimizing the excess utilization of procedures, focusing on quality rather than quantity of interventions. We identified specific patient, physician, and hospital factors associated with poor outcomes for inpatients undergoing cranial neurosurgical procedures. In addition, we investigated the association of the intensity of neurosurgical care, as a measure of the aggressiveness of intervention, with specific outcomes tracked by the Centers for Medicare and Medicaid Services (CMS).

At the patient level, we demonstrated that increasing age, male gender, worse general health, low income, coverage by Medicaid, and minority racial status were associated with higher in-hospital neurosurgical mortality, increased rates of unfavorable discharge, and prolonged length of stay. These are all important factors previously described as significant contributors to worse outcomes, and their identification validates our model. In addition, we observed that patients without insurance coverage were associated with higher mortality, increased LOS, and decreased rate of unfavorable discharge. This observation could represent the lower level of health of this group. It also reflects the difficult disposition of this patient population, necessitating prolonged hospitalizations to facilitate their home transition.

Hospital-level factors were also significant. Institutions located in the Northeast were associated with higher mortality, increased rate of unfavorable discharge, and prolonged LOS. A significant concentration of academic institutions, which function as referral centers are located in the Northeast. The observed associations likely reflect the increased complexity of the patients treated by these facilities.

We introduced intensity of neurosurgical care, as a new metric of general practice patterns in neurosurgery. It reflects the aggressiveness of operative intervention for all neurosurgical pathologies. We did not observe an association of increasing intensity of neurosurgical care, for similar patients, with mortality throughout the United States. In addition, no such association was observed with rates of unfavorable discharge and length of stay. Although the performance of more neurosurgical procedures per capita, is associated with higher cost, it does not seem to correlate with improved outcomes.

Although provider volume has been demonstrated to have a beneficial effect on survival for several surgical procedures [20], [21], there is no such correlation with the intensity of neurosurgical intervention at the state level. The former association has been attributed to the positive effect of surgeon experience on the postoperative complication rate. On the contrary, the lack of correlation observed in the present study is more reflective of the practice patterns in a state, and corresponds to the aggressiveness of providers in an area, without necessarily correlating with their experience.

Our study provides limited guidance on the potential impact of reducing regional disparities in utilization. From a clinical perspective, it is important to recognize that this analysis does not address the question of how the amount of care for an individual patient in a specific case would affect the patient's clinical outcome. From a policy perspective, our study does not indicate whether it is possible to reduce overutilization and spending without affecting patient outcomes. However, if the United States as a whole could safely achieve intensity levels comparable to those of the lowest-utilizing regions, significant savings could be achieved. Further research in that direction is needed.

The present study has several limitations common to administrative databases. First, indication bias and residual confounding could account for some of the observed associations. Second, some coding inaccuracies will undoubtedly occur and can affect our estimates. This is no different than other studies involving the NIS. Third, the NIS during the years studied did not include hospitals from all states. However, the hospitals included were still diverse with respect to size, region, and academic status, supporting the generalizability of our findings. Fourth, in order to estimate the number of procedures performed per state we used the standard weights provided by HCUP. Although these calculations are not expected to be absolutely accurate, they are adequate for the stratification of intensity of care we used in this analysis.

Fifth, the observed differences can be attributed to differences in the patient populations in separate geographic regions. Our risk adjustment aimed at minimizing this bias, so that the observed comparisons would be applied on similar patients. The identification of well-established risk factors contributing to higher mortality is additionally validating our models. Sixth, the NIS does not provide any information on the post-acute care of the patients, or whether the cases described were elective or emergent. Seventh, we used largely ecologic data, and therefore causality cannot be established based on this data.

## Conclusions

Practice patterns vary widely throughout the United States for multiple surgical interventions. We observed significant disparities in the intensity of neurosurgical care in the United States. Increased intensity was not associated with mortality, rate of unfavorable discharge, and length of stay for neurosurgical procedures. This observation supports the need for emphasis on accountability through minimizing the excess utilization of procedures, while focusing on quality rather than quantity of interventions.

## Supporting Information

Table S1 **Coding definitions.**
(DOC)
