# Supplementary material for: The Association of Unfavorable Outcomes with the Intensity of Neurosurgical Care in the United States
Source: PLoS One. 2014 Mar 19;9(3):e92057. doi: 10.1371/journal.pone.0092057 (PMC3960180; doi:10.1371/journal.pone.0092057)
Supplement: Table S1 — Coding definitions. (DOC) [file pone.0092057.s001.doc]

**The association of unfavorable outcomes with the intensity of neurosurgical care in the United States**

Symeon Missios, M.D. ^1^,*, Kimon Bekelis, M.D. ^2^,*

^1^ Department Neurosurgery, Cleveland Clinic, Cleveland, OH

^2^ Section of Neurosurgery, Dartmouth-Hitchcock Medical Center, Lebanon, NH

**Table S1.** Coding definitions

| **Table S1. Coding definitions** | | | | |
| --- | --- | --- | --- | --- |
| **GROUP** | **CATEGORY** | **Variable** | **CODES** | **NOTES/CODING RULES** |
| **Procedures** | **Craniotomy for aneurysm clipping** | P1 | **39.51** | *ICD-9 Procedure codes* |
|  | **Craniotomy for tumor, epilepsy and AVM resection** | **P3** | **01.53**  **01.59**  **01.51 (meningiomas)** | *ICD-9 Procedure codes* |
|  | **Brain biopsy** | **P4** | **01.11**  **01.12 01.13**  **01.14** | *ICD-9 Procedure codes* |
|  | **Craniotomy/burr hole for trauma** | **P5** | **01.2**  **01.21**  **01.22**  **01.23**  **01.24**  **01.25**  **01.26**  **01.27**  **01.28**  **02.02** | *ICD-9 Procedure codes* |
|  | **Shunt Placement** | **P8** | **02.2**  **02.32**  **02.33**  **02.34**  **02.35**  **02.39**  **03.7**  **03.71**  **03.72**  **03.79**  **02.4**  **02.41**  **02.42**  **02.43** |  |
| **Procedures** | **Deep brain stimulation** | **P9** | **02.93 (leads)**  **01.22**  **If multiple DBS codes in an admission then consider patient as one procedure** |  |
|  | **Additional codes** |  | **01.31**  **01.32**  **01.39**  **01.41**  **01.42**  **01.52**  **02.0**  **02.05**  **02.07**  **02.02**  **04.01**  **04.41**  **07.61**  **07.62**  **07.64**  **07.65** |  |
